# Mutations in Pseudohypoparathyroidism 1a and Pseudopseudohypoparathyroidism in Ethnic Chinese

**DOI:** 10.1371/journal.pone.0090640

**Published:** 2014-03-20

**Authors:** Yi-Lei Wu, Daw-Yang Hwang, Hui-Pin Hsiao, Wei-Hsin Ting, Chi-Yu Huang, Wen-Yu Tsai, Hung-Chun Chen, Mei-Chyn Chao, Fu-Sung Lo, Jeng-Daw Tsai, Stone Yang, Shin-Lin Shih, Shuan-Pei Lin, Chiung-Ling Lin, Yann-Jinn Lee

**Affiliations:** 1 Department of Pediatrics, Mackay Memorial Hospital, Taipei, Taiwan; 2 Department of Pediatrics, Changhua Christian Hospital, Changhua, Taiwan; 3 Division of Nephrology, Department of Medicine, Kaohsiung Medical University Hospital, Kaohsiung, Taiwan; 4 Department of Pediatrics, Kaohsiung Municipal Hsiao Kang Hospital, Kaohsiung Medical University, Kaohsiung, Taiwan; 5 Department of Pediatrics, National Taiwan University Hospital, Taipei, Taiwan; 6 Faculty of Renal Care, Department of Medicine, College of Medicine, Kaohsiung Medical University, Kaohsiung, Taiwan; 7 Division of Genetics, Endocrinology and Metabolism, Department of Pediatrics, Kaohsiung Medical University Hospital, Kaohsiung, Taiwan; 8 Genome Medicine, College of Medicine, Kaohsiung Medical University, Kaohsiung, Taiwan; 9 Department of Pediatrics, Chang Gung Memorial Hospital, Taoyuan, Taiwan; 10 College of Medicine, Chang Gung University, Taoyuan, Taiwan; 11 Mackay Junior College of Medicine, Nursing, and Management, New Taipei City, Taiwan; 12 Department of Urology, Mackay Memorial Hospital, Taipei, Taiwan; 13 Department of Radiology, Mackay Memorial Hospital, Taipei, Taiwan; 14 Department of Medical Research, Mackay Memorial Hospital, Taipei, Taiwan; 15 Department of Infant and Child Care, National Taipei University of Nursing and Health, Taipei, Taiwan; 16 College of Medicine, Taipei Medical University, Taipei, Taiwan; 17 Institute of Biomedical Sciences and Department of Medicine, Mackay Medical College, New Taipei City, Taiwan; Odense University hospital, Denmark

## Abstract

An inactivating mutation in the *GNAS* gene causes either pseudohypoparathyroidism 1a (PHP1A) when it is maternally inherited or pseudopseudohypoparathyroidism (PPHP) when it is paternally inherited. We investigated clinical manifestations and mutations of the *GNAS* gene in ethnic Chinese patients with PHP1A or PPHP. Seven patients from 5 families including 4 girls and 2 boys with PHP1A and 1 girl with PPHP were studied. All PHP1A patients had mental retardation. They were treated with calcitriol and CaCO3 with regular monitoring of serum Ca levels, urinary Ca/Cr ratios, and renal sonography. Among them, 5 patients also had primary hypothyroidism suggesting TSH resistance. One female patient had a renal stone which was treated with extracorporeal shockwave lithotripsy. She had an increased urinary Ca/Cr ratio of 0.481 mg/mg when the stone was detected. We detected mutations using PCR and sequencing as well as analysed a splice acceptor site mutation using RT-PCR, sequencing, and minigene construct. We detected 5 mutations: c.85C>T (Q29*), c.103C>T (Q35*), c.840-2A>G (R280Sfs*21), c.1027_1028delGA (D343*), and c.1174G>A (E392K). Mutations c.840-2A>G and c.1027_1028delGA were novel. The c.840-2A>G mutation at the splice acceptor site of intron 10 caused retention of intron 10 in the minigene construct but skipping of exon 11 in the peripheral blood cells. The latter was the most probable mechanism which caused a frameshift, changing Arg to Ser at residue 280 and invoking a premature termination of translation at codon 300 (R280Sfs*21). Five *GNAS* mutations in ethnic Chinese with PHP1A and PPHP were reported. Two of them were novel. Mutation c.840-2A>G destroyed a spice acceptor site and caused exon skipping. Regular monitoring and adjustment in therapy are mandatory to achieve optimal therapeutic effects and avoid nephrolithiasis in patients with PHP1A.

## Introduction

Albright hereditary osteodystrophy (AHO; OMIM #103580) was described by Albright and Smith in 1942 [1]. It is characterized by short stature, round facies, brachydactyly, and short fourth and fifth metacarpals, metatarsals, or both. Pseudohypoparathyroidism (PHP) includes a heterogeneous group of metabolic disorders characterized by hypocalcemia, hyperphosphatemia, and an elevated PTH level because of PTH resistance [2]. On the basis of the presence or absence of AHO, urinary cAMP response to PTH infusion, resistance to other peptide hormones, and diminished in vitro Gsα activity, PHP is categorized into pseudohypoparathyroidism 1a (PHP1A; OMIM #103580), pseudohypoparathyroidism 1b (PHP1B; OMIM #603233), pseudohypoparathyroidism 1c (PHP1C; OMIM #612462), and pseudohypoparathyroidism 2 (PHP2; OMIM %203330) [3], [4], [5]. Patients with pseudopseudohypoparathyroidism (PPHP; OMIM #612463) have AHO but no resistance to PTH or other hormones.

Genetic mutations for the different subtypes of PHP involve the α-subunit of the stimulatory G protein (Gsα) which is encoded by the *GNAS* complex locus located on chromosome 20q13.11 [6]. Gsα expression is biallelic in most tissues, however, only maternal allele is preferentially expressed in renal proximal tubules, pituitary, thyroid, and gonads [5]. Therefore inactivating *GNAS* mutations on either the paternal or maternal allele result in Gsα deficiency leading to AHO [7] but resistance of target organs to PTH and other hormones which act through cAMP only if the mutations are on the maternal allele [8]. Four additional imprinted gene products from the *GNAS* complex locus are paternally expressed XLαs, A/B (also referred as 1A) and antisense transcripts (GNASAS), and maternally expressed NESP55 transcript [9], [10], [11].

PHP1A is caused by mutations in the *GNAS* gene on the maternal allele, whereas PPHP is caused by mutations in the gene on the paternal allele [4]. Thus PPHP and PHP1A can occur in different generations of the same family. The clinical presentation of PHP1C is similar to PHP1A except normal in vitro Gsα activity [5]. Mutations in PHP1C are all in the C-terminal region of Gsα (p.L388P, p.L388R, p.Y391*, p.E392K, and p.E392*) and disrupt receptor-mediated activation but display normal receptor-independent activation [4], [12]. The molecular defects of familial autosomal dominant PHP1B can be due to microdeletions on the maternal allele of the *STX16* gene or *NESP55* gene and/or antisense exons 3 and 4, or paternal uniparental isodisomy [9], [13], [14], [15], [16], [17]. These variations lead to loss of methylation in exon A/B differentially methylated region (DMR), diminishing maternal expression of Gsα in renal proximal tubules [9]. However, most PHP1B are sporadic and their disease causing genes remained to be identified [18]. The genetic cause of PHP2 is unknown. The similarity in urinary excretion of cAMP following PTH administration between acrodysostosis with mutations in *PRKAR1A* or *PDE4D* and PHP2 indicates that genes other than *GNAS* may be responsible for PHP2 [19], [20], [21].

Although 176 *GNAS* mutations have been reported in the Human Gene Mutation Database (HGMD, http://www.hgmd.org/; searched on 2013/08/27) and Leiden Open Variation Database (LOVD, http://www.lovd.nl; searched on 2013/08/27) [22], few reports are on Asians [23], [24], [25]. We conducted clinical and molecular investigations on ethnic Chinese patients with PHP1A or PPHP and compared the findings with those reported in patients of other ethnicities. We also assessed the effect of a novel splice acceptor site mutation using a minigene construct and mRNA analysis.

## Materials and Methods

### Patients

We studied 7 patients from 5 families. These patients included 4 girls and 2 boys with PHP1A and 1 girls with PPHP, diagnosed at 5.8–13.2 years of age. The diagnosis of PHP1A was based on the following criteria: features of AHO, hypocalcemia, hyperphosphatemia, and resistance to PTH [2]. The diagnosis of PPHP was based on the presence of AHO without biochemical or hormonal abnormalities [2]. Obesity was defined as a BMI value of >95th percentile according to the age- and gender-specific standards for ethnic Chinese children in Taiwan [26]. Patients 1A and 1B are siblings, as are patients 2A and 2B. The institutional review board of Mackay Memorial Hospital approved this study and all subjects including their parents or guardians gave written informed consent.

### Detection of Mutations in the *GNAS* Gene

Genomic DNA from peripheral blood leukocytes of patients and their relatives was analyzed. All 13 exons and intron–exon boundaries of the *GNAS* gene were amplified by PCR, using primers and conditions described by Mantovani et al [27]. PCR products were confirmed by electrophoresis on 1.5% agarose gels and were sequenced by using an ABI 3730XL DNA Analyzer (Applied Biosystems, Foster City, CA, USA). The GNAS mRNA and protein reference sequences were NM_000516.4 and NP_000507.1, respectively.

### Minigene Constructs, Cell Culture, and Transient Transfection

**Figure 1:**
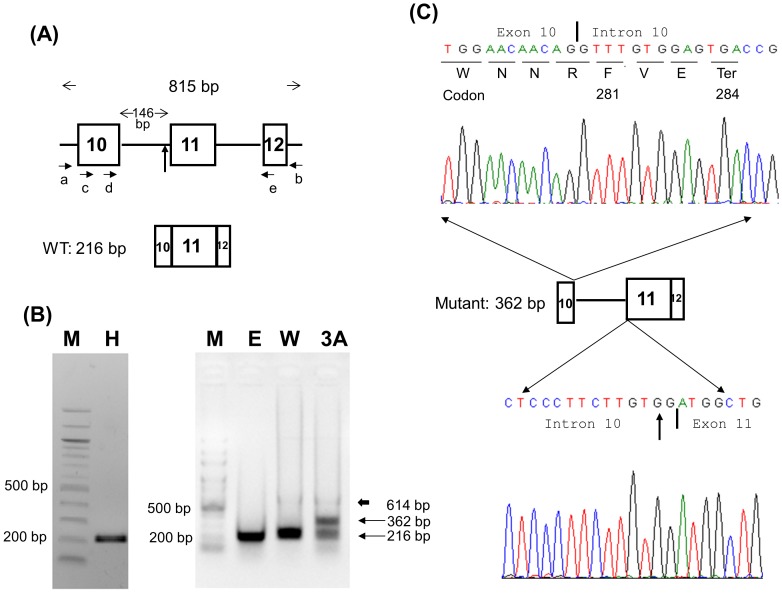
Minigene analysis of c.840-2A>G mutation in patient 3A. (A) The GNAS-IVS10 minigene and primer designs. The construct made by using primers a and b spans from introns 9 to 12 and contains exons 10, 11, and 12 with a total length of 815 bp. Primers c, d, and e were used for semi-nested PCR. (B) COS-7 cells were used for transient transfection study. An extra 362-bp band was found in the mutant (lane 3A) compared with a single 216-bp band in the wild type vectors (lane W). The 362-bp band in lane 3A was subcloned and sequenced. The electropherogram is shown in Panel C. The thick arrow indicates a faint 614-bp band amplified from DNA carried over from COS-7 cells or mini-gene plasmids. Lane H, healthy individual; M, 100 bp DNA ladder; E, empty pcDNA3.1 vector; W, IVS10 wild minigene; 3A, IVS10 mutant minigene. (C) TOPO TA subcloning and sequencing reveal the 362 bp band containing the complete intron 10 of 146 bp. The inclusion of intron 10 changes amino acid Trp to Phe at residue 281 and causes an earlier termination of translation at codon 284 (p.Trp281PhefsTer4 or p.W281Ffs*4). The vertical arrows in Panels A and C denote the site of the c.840-2A>G mutation. The vertical bars in Panel C indicate the exon-intron or intron-exon boundaries.

To analyze the effect of the c.840-2A>G mutation at the splice acceptor site of intron 10 on splicing in patient 3A, a GNAS-IVS10 minigene was constructed. Briefly, 100 ng of genomic DNAs from control individual and patient 3A were used as PCR templates. Primers a, 5′-TGTTAGGGATCAGGGTCGCTG-3′ (located in intron 9), and b, 5′-AGAGGAGGAACAAGAGAGGAA-3′ (located in intron 12), were designed to amplify an 815 bp region (Figure 1A). AccuPrime™ Taq DNA Polymerase High Fidelity (Invitrogen, Carlsbad, CA, USA) was used according to the manufacturer’s protocol with an initial denaturation at 94°C for 30 seconds, followed by 30 cycles of denaturation (94°C for 15 second), annealing (60°C for 15 seconds), and extension (68°C for 1 minute). The 815 bp PCR products were electrophoresed on 1.5% agarose gels with 100 bp DNA ladder to confirm the correct size, and then purified with QIAquick Gel Extraction Kit (QIAGEN, Valencia, CA, USA). The purified PCR products were subcloned into the pcDNA™3.1/V5-His vector by using pcDNA™3.1/V5-His TOPO® TA Expression Kit (Invitrogen, Carlsbad, CA, USA) according to the manufacturer’s protocol. Both wild-type and mutant minigene constructs were confirmed by Sanger sequencing to ensure correct insert direction and sequences.

COS-7 cells were from ATCC (American Type Culture Collection, CRL-1651) and cultured in DMEM with 10% fetal bovine serum (FBS) plus antibiotics penicillin and streptomycin. Transient transfection was performed at ∼80% confluence and 10% FBS/DMEM was replaced with Opti-MEM® reduced serum medium (Life Technologies) before transfection. One μg of plasmid (containing either wild-type or mutated GNAS-IVS10 minigene) in 100 μl Opti-MEM® was mixed with 3 μl of FuGENE6 (Roche, Indianapolis, IN, USA) before adding to a well of a 6-well plate. Opti-MEM® was removed and replaced with 10% FBS/DMEM 6 hours after transfection.

### RT-PCR and Semi-nested PCR

Transfected COS-7 cells were harvested 24 h after transfection and total RNA was extracted with the standard Trizol method (Invitrogen, Carlsbad, CA, USA). Reverse-transcription (RT) of the total RNA was performed by using M-MLV RT kit (Promega, Madison, WI, USA) and the cDNA product was amplified with semi-nested PCR by using primers c, 5′-GGTGGCCAGCAGCAGCTACA-3′, d, 5′-CGCCTGCAGGAGGCTCTGAAC-3′, and e, 5′-CCGGGTCACGCGTGGGTC-3′ (Figure 1A). In the semi-nested PCR, primers c and e were used for the first PCR. The PCR product was diluted 100-fold and then 1 μl of the diluted PCR product was used for second PCR with primers d and e. The RT-PCR products were gel purified and subcloned into pCR2.1 plasmids with pCR2.1 TOPO TA Cloning Kit (Invitrogen, Carlsbad, CA, USA) and Sanger sequenced.

### RT-PCR of the Peripheral Blood Leukocytes

**Figure 2:**
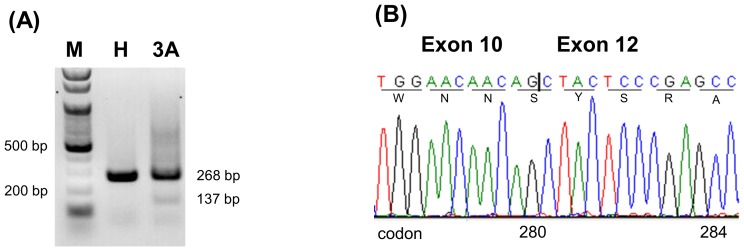
RT-PCR analysis on the RNA from the peripheral blood leukocytes of a healthy control and patient 3A with primers c and e. (A) The electropherogram shows a band of 268 bp in the healthy control (lane H) but two bands, 268 bp and 137 bp, in patient 3A (lane 3A). The 137-bp band is faint. M indicates 100 bp DNA ladder; H, healthy control; 3A, patient 3A. (B) Sequence analysis of the 137-bp band reveals no exon 11. The deletion of exon 11 results in a frameshift changing Arg to Ser at residue 280 and causing an earlier termination of translation at codon 300 (p.Arg280SerfsTer21 or p.R280Sfs*21). The vertical bar indicates the boundary of exons 10 and 12.

Total RNA from the peripheral blood leukocytes of a healthy control and patient 3A was extracted with the same standard Trizol method and reverse-transcribed with the same M-MLV RT kit (Promega, Madison, WI, USA). Then the cDNA product was amplified with primers c and e (Figure 1A). The PCR product was electrophoresed on a 1.5% agarose gel (Figure 2A).

## Results

### Clinical Manifestations

**Figure 3:**
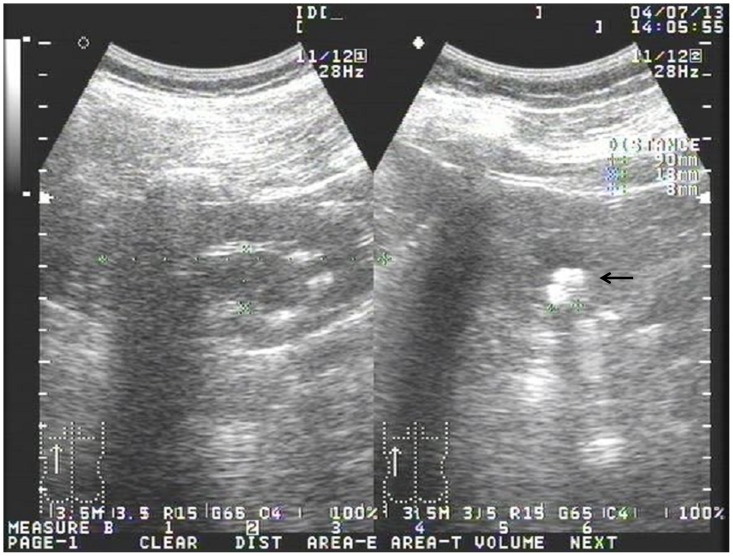
Sonography of the left kidney. A hyperechoic mass measuring 8(right panel, arrow).

**Figure 4:**
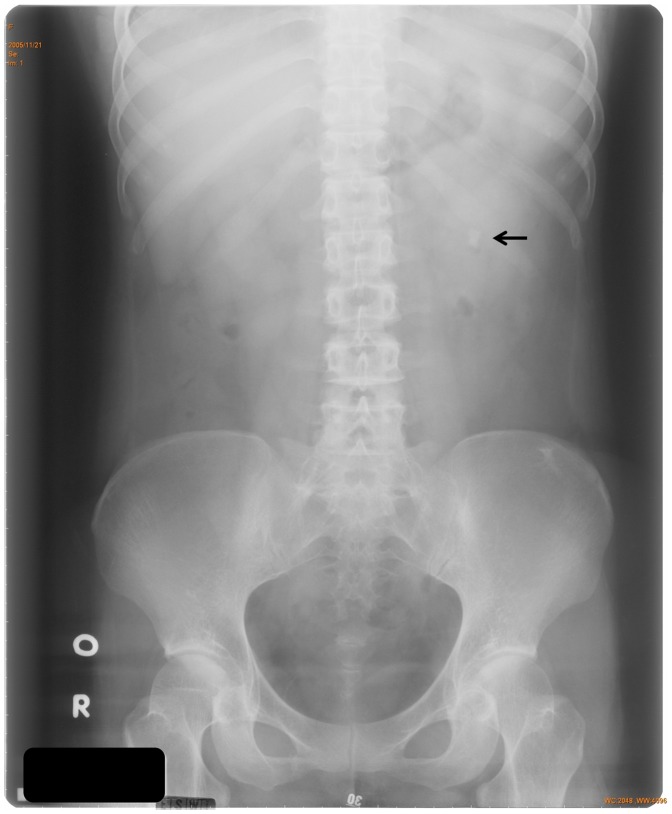
Radiography of the kidneys and urinary bladder. A radio-opaque stone measuring 11×6 mm in size in the central portion of the left kidney (arrow).

All 7 patients had features of AHO (Table 1). Four (1A, 2A, 2B, and 4A; 66.7%) of 6 PHP1A patients were obese. The patients with PHP1A received treatment of calcitriol and calcium carbonate (CaCO_3_) starting at diagnosis. Laboratory parameters were monitored every 3–6 months and renal sonography every year. The dosages of calcitriol and CaCO_3_ were adjusted according to serum calcium levels and urinary Ca/Cr ratio. Patient 3A took calcitriol (10 ng/kg/day, twice a day) and CaCO_3_ (elemental Ca 20 mg/kg/day, 4 times a day). Ossification in soft tissue of the sole of her right foot was noted and excised at 17.5 years of age. A renal stone with hyperechogenicity was detected by sonography (Figure 3) when she was 23.4 years old after 8.9 years of therapy. The stone was radio-opaque by radiography (Figure 4). Her serum total Ca levels had been between 2.0 and 2.3 mmol/l (ionized Ca levels between 1.1 and 1.2 mmol/l, reference 1.20–1.38 mmol/l) with few occasions of hypocalcemia (the lowest ionized Ca level 1.08 mmol/l) due to inadequate compliance. Urinary Ca/Cr ratios had been between 0.013 and 0.125 (reference <0.213 mg/mg [10]) except it was 0.307 mg/mg 4 months before and 0.481 mg/mg at the detection of the renal stone when she was taking calcitriol 10.2 ng/kg/day and elemental Ca 20.5 mg/kg/day. The dose of CaCO_3_ was immediately decreased to 16.8 mg/kg/day of elemental Ca and a follow-up urinary Ca/Cr was 0.071 mg/mg. The stone was disintegrated with extracorporeal shockwave lithotripsy.

**Table 1:** Characteristics of patients with pseudohypoparathyroidism 1a (PHP1A) or pseudopseudohypoparathyroidism (PPHP).

Family	1	1	2	2	3	4	5	Reference
Member	1A	1B	2A	2B	3A	4A	5A	
Diagnosis	PHP1A	PHP1A	PHP1A	PHP1A	PHP1A	PHP1A	PPHP	
Age at diagnosis (year)	12.2	9.3	8.8	5.8	14.5	13.0	13.2	
Sex	Female	Female	Female	Male	Female	Male	Female	
Round facies	+	+	+	+	+	+	+	
Short thick neck	+	+	+	+	+	+	–	
Short 4th and 5th metacarpals	+	+	+	+	+	+	+	
Short 4th and 5th metatarsals	+	+	+	+	+	+	+	
Brachydactyly	+	+	+	+		+		
BMI (kg/m^2^)	24.1^a^	15.7	25^a^	21.6^a^	21.2	36^a^	21.7	
Short stature	+	+	+	+	+	+	+	
Subcutaneous ossification	–	–	+	+	+	+	–	–
Intelligence quotient	68	62	59	68	44	57	Average^b^	90–109
Ca (mmol/L)	2.1	1.4	1.65	2.4	1.6	1.5	2.25	2.2–2.6
P (mmol/L)	1.73	2.3	2.2	2.0	2.56	3.1	1.55	0.8–1.44
Alkaline phosphatase (IU/L)	180	447			215	410	96	105–420
Intact-PTH (pmol/L)	28.7	97.5	57.5	68.6	26.4	21.69	2.88	1.1–7.2
Free-T4 (pmol/L)	3.9	11.9	15.8	11	12.4	17.1	19	10.2–25.7
TSH (mIU/L)	28.4	15.25	46	4.12	10.71	10.37	2.81	0.54–4.58
LH (IU/L)	7.6	<3.8	17.3		29.25	5.07	3.8	2–9
FSH (IU/L)	12.94	8.88	57.9		28	2.78	3.11	1.5–12.4
E2 (pmol/L)	60	36	213		73		419	91–355
Prolactin (pmol/L)	247	180			385			<652
Menarche (yr)	12.25	13	13		11.5		11.33	
GNAS mutation								
DNA level	c.85C>T	c.85C>T	c.103C>T	c.103C>T	c.840-2A>G	c.1027_1028delGA	c.1174G>A	
Protein level	p.Gln29Ter	p.Gln29Ter	p.Gln35Ter	p.Gln35Ter	p.Arg280SerfsTer21	p.Asp343Ter	p.Glu392Lys	
1-letter symbol	p.Q29*	p.Q29*	p.Q35*	p.Q35*	p.R280Sfs*21	p.D343*	p.E392K	

Patients 1A and 1B and patients 2A and 2B are siblings.

a BMI >95th percentile.

b The performance in school was average.

Five (83.3%) PHP1A patients also had primary hypothyroidism with elevated thyroid stimulatory hormone (TSH) levels and low or normal free T4, suggesting TSH resistance at diagnosis. All PHP1A girls had menarche at the normal age (11.5–13 years), but patient 1A had menstrual irregularity and needed progesterone supplement to induce menstruation at age 14 years. All PHP1A patients had mental retardation, with IQs of 44–68. The PPHP patient had not taken an IQ test, but her performance in school was average.

### *GNAS* Mutations

**Figure 5:**
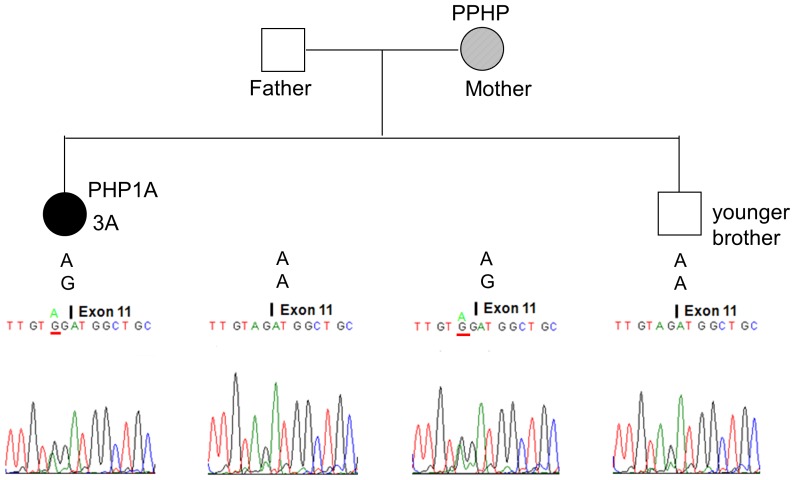
Pseudohypoparathyroidism 1a (PHPIA) caused by the c.840-2A>G mutation in Family 3. The mutant c.840-2A>G allele of patient 3A (solid circle) was inherited from her mother (hatched circle) who had PPHP. Her father and younger brother were normal. The genotype of each member at this site is shown above the electropherogram.

**Figure 6:**
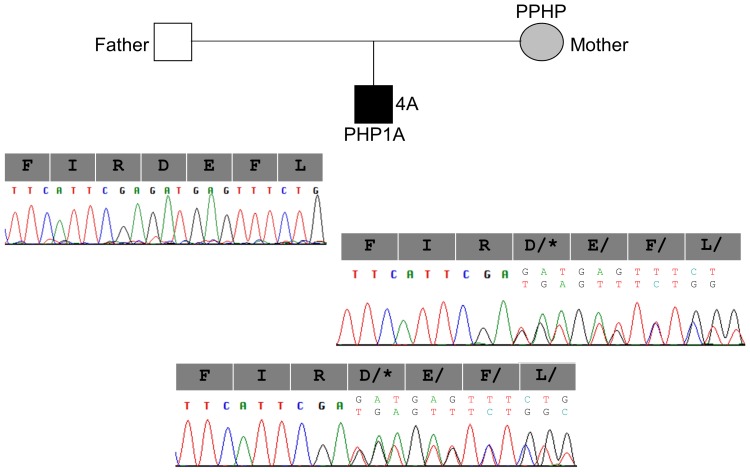
Pseudohypoparathyroidism 1a (PHPIA) caused by the c.1027_1028delGA mutation in Family 4. The mutant c.1027_1028delGA allele of patient 4A (solid square) was inherited from his mother (hatched circle) who had pseudopseudohypoparathyroidism (PPHP). His father was normal. The deletion of two nucleotides in the mutant allele causes a frameshift resulting in a premature termination of translation at codon 343 (p.Asp343Ter or p.D343*). The genotype of each member at this site is shown above the electropherogram.

A total of 5 heterozygous mutations were identified in 5 families: c.85C>T (p.Q29*), c.103C>T (p.Q35*), c.840-2A>G (p.R280Sfs*21), c.1027_1028delGA (p.D343*), and c.1174G>A (p.E392K) (Table 1). Mutations c.840-2A>G and c.1027_1028delGA were novel, whereas the others have been reported in patients with PHP1A [2]. The mutant c.840-2A>G allele of patient 3A (Figure 5) and c.1027_1028delGA allele of patient 4A (Figure 6) were passed from their mothers who had PPHP.

### Minigene Constructs, RT-PCR, and Semi-nested PCR

Using the GNAS-IVS10 minigene model, we found that the c.840-2A>G mutation destroyed the splicing acceptor and caused the retention of intron 10, resulting in a 362-bp band and a frameshift (Figure 1). This changed the amino acid from Trp to Phe at residue 281, followed by a premature termination of translation at codon 284 (p.W281Ffs*4). Sequencing of the PCR band from lane E (mock transfection with empty vector) in Figure 1B showed the same length of 216 bp similar to human *GNAS* sequence, except 2 variants of c.864C>T and c.885T>C (data not shown).

### RT-PCR of Peripheral Blood Leukocytes

In contrast to the results from the minigene model, RT-PCR of the RNA from the peripheral blood cells of patient 3A revealed only a 268-bp band and a faint but definite 137-bp band (Figure 2A). Sequencing confirmed the shorter PCR fragment containing no exon 11 (Figure 2B). The deletion of exon 11 caused a frameshift changing Arg to Ser at residue 280 and resulting in an earlier termination of translation at codon 300 (p.R280Sfs*21).

## Discussion

### Pathogenicity of the Detected Mutations

We detected 5 mutations in patients with either PHP1A or PPHP from 5 ethnic Chinese families and all of them co-segregated with disease status in each family. Among them, c.840-2A>G and c.1027_1028delGA were novel. The two mutations were not found in the HGMD, LOVD and NCBI SNP Databases, and 1000 Genomes Projects. They caused frameshifts resulting in premature terminations of translation.

### Genetic Epidemiology of PHP

The prevalence of PHP is largely unknown except a reported prevalence of 3.4 (95% CI, 2.6–4.2) per million from Japan [28]. The other estimated prevalence is 0.79 per 100,000 (according to Orphanet Report Series, November 2008) described in a recent publication [9]. A total of 17 different mutations have been identified in 24 PHP1A and 4 PPHP individuals from Asia [23], [24], [25], [29], [30], [31], [32]. Our series added five different *GNAS* mutations including two novel ones to the list and increased the number to 22. More than half (13 out of 22) of these mutations have not been reported in the other part of the world. And 24% (6 out of 22) are located in exon 1. Mutations c.565_568delGACT, c.308T>C, and c.348_349insC were found in 3, 2, and 2 families. The remaining mutations were reported in only one family each. The trend is similar to those in recently published cohort studies [33], [34].

### Gsα Protein

Parathyroid hormone (PTH) stimulates the formation of intracellular cyclic adenosine monophosphate (cAMP) by adenylyl cyclase via the activation of the Gs protein which is bound to the intracytoplasmic portion of the PTH/PTHrP receptor [35]. The Gs protein is heterotrimeric and composed of α, β, and γ subunits. A guanosine diphosphate (GDP) is bound to the Gsα subunit in the inactive state. Upon PTH binding to the PTH/PTHrP receptor, a guanosine triphosphate (GTP) replaces the GDP. The Gsα-GTP complex is released from the receptor and the βγ subunits and then activates adenylate cyclase which catalyzes the formation of cAMP. The cAMP activates protein kinase A (PKA) which causes phosphaturia. The intrinsic guanosine triphosphatase activity of the Gsα subunit hydrolyzes bound GTP to GDP and terminates the signal transduction. By coupling to the 7-transmembrane-domain receptors, the Gsα subunit is involved in signal transduction of several extracellular messengers and diverse intracellular effector pathways [3]. The signal-dependent manner of the Gα subunit in binding guanine nucleotide confers specificity to each G protein.

### Variability of the Phenotype

The diagnostic age of our small cohort is not bimodally distributed as those in other reports [34], [36] because our patients were recruited only from pediatric departments and presented with AHO phenotypes instead of hypocalcemia-related symptoms. Most of our patients had all manifestations of AHO phenotype except patients 3A and 5A who did not have brachydactyly. Families 2, 3 and 4 presented with subcutaneous ossification but no sign of progression to progressive osseous heteroplasia. No apparent delayed puberty were found in female individuals according to their menarche age. Many PHP1A and PPHP patients have a similar heterozygous loss-of-function mutation in the *GNAS* gene, however, the severity of AHO is variable [9]. Two siblings (1A and 1B) of family 1 with Q29* mutation inherited from the mother had the features of AHO and multiple hormone resistance. However, the mother had only mild brachydactyly and relative short stature compared with her sisters. The intrafamilial or interfamilial variability of a phenotype could be due to epigenetic alterations [37], altered transcriptional regulation [38], or effects of other genes [39], [40].

### Mutations at Splice Sites

Mutations at splice sites cause intron retention, exon skipping, or activation of a cryptic splice site resulting in partial retention of introns or partial loss of exons [3], [41]. The analyses of the c.840-2A>G mutation showed inconsistent results from different methods. The mutant allele was expressed with retained intron 10 in the minigene model, however, no mRNA with retention of intron 10 was detected in the patient’s peripheral blood cells. On the contrary, deletion of exon 11 was found in the peripheral blood cells but not in the minigene model. The COS-7 cell transfection experiment was affected by the endogenous *Gnas* from *Chlorocebus aethiops* (African green monkey) because Sanger sequencing showed 2 variants (corresponding to the human GNAS position c.864C>T and c.885T>C) which were not in our design. The best cell model should be of null GNAS, such as the Gnas E2−/E2− cells from the mouse [42].

Different splicing results caused by the c.840-2A>G mutation can be due to cell-specific GNAS expression in the transfected COS-7 cell and nucleated blood cell using different *trans*-activating factors. Recognition of exon-intron splice site has been shown to be influenced by the upstream introns and splicing signals in the minigene system [43], [44]. It is also possible that the mRNA with retention of intron 10 was expressed in the peripheral blood cells but degraded through nonsense-mediated decay [45] to a level which was too low to be detected by our method. We could not know which aberrant GNAS mRNA transcript existed in the renal tubule since the patient did not donate her renal tissue. This mRNA splicing discrepancy between *in vitro* and *in vivo* systems have also been observed in other genes [46], [47]. Based on the expression of the mutated GNAS gene in the patient’s leukocytes, we concluded that the c.840-2A>G mutation most probably caused deletion of exon 11, resulting in a frameshift changing Arg to Ser at residue 280 and invoking a premature termination of translation at codon 300 (p.Arg280SerfsTer21) (Figure 2). Quantitative PCR on patient’s EBV-transformed lymphoblasts treated with cycloheximide as a nonsense-mediated decay inhibitor can elucidate the mechanism of low expression level of this splice site mutation [48], [49].

### Mutations in the Maternal Allele of the *GNAS* Gene

All of our PHP1A patients had mental retardation and 80% had primary hypothyroidism with elevated TSH levels. Our findings corroborate previous reports showing that hypothyroidism was present in the majority of patients even at their initial presentation [12], [50], [51]. The *GNAS* gene is biallelically expressed in most tissues, but the paternal allele is variably expressed in the proximal renal tubules, thyroid gland, gonads, and pituitary gland [5]. Therefore mutation in the maternal allele results in hypocalcemia, hyperphosphatemia, hypophosphaturia, resistance to TSH and gonadotropins in PHP1A patients. The variable expression of the paternal allele in the thyroid might be responsible for the wide spectrum of thyroid function alterations in PHP1A patients [52], [53] as in our patients.

### Hypercalciuria in PHP1A

In the kidney, most filtered calcium is paracellularly reabsorbed in the proximal tubule and the rest is transcellularly reabsorbed [9], [54]. PTH stimulates the production of 25(OH)D 1α-hydroxylase and inhibits phosphate reabsorption in the proximal tubule as well as promotes reabsorption of calcium in the distal tubule [54]. In patients with PHP1A, the proximal tubule does not respond to PTH whereas the distal tubule does [55]. Phosphaturic effect of PTH is defective but anticalciuric action remains functional, therefore renal stones are rare in PHP1A patients [9]. However, a renal stone developed in patient 3A after 8.9 years of calcitriol and CaCO_3_ therapy when she had hypercalciuria on a dose of calcitriol lower than the recommended range of 15–30 ng/kg/day [54] and intake of elemental Ca estimated to be at the upper limit of the recommended range of 20–32.5 mg/kg/day [56] (20.5 mg/kg/day of supplementary Ca and estimated 11 mg/kg/day from diet [57]). This suggested that renal stones could occur in a PHP1A patient during a period of hypercalciuria. Thus the dosage of calcitriol and elemental Ca should be individualized to maintain normalized PTH and serum calcium levels without hypercalciuria [5].

### Conclusions

We report 5 *GNAS* mutations in ethnic Chinese patients with PHP1A or PPHP from 5 families and expanded the spectrum of mutations with 2 novel ones (c.840-2A>G and c.1027_1028delGA). Clinically diagnosis of PHP is straightforward and molecular diagnosis is powerful to elucidate the genetic causes for counseling in affected families. Regular monitoring and adjustment in therapy are mandatory to achieve optimal therapeutic effects and avoid nephrolithiasis in patients with PHP1A.
